# Effect of replacing whole wheat with broken rye as a sustainable grain in diets of fattening turkeys on growth performance, litter quality, and foot pad health

**DOI:** 10.3389/fvets.2023.1142500

**Published:** 2023-04-11

**Authors:** Jan Berend Lingens, Christian Visscher, Christian Sürie, Richard Grone, Andreas von Felde, Volker Wilke, Amr Abd El-Wahab

**Affiliations:** ^1^Institute for Animal Nutrition, University of Veterinary Medicine Hannover, Hannover, Germany; ^2^Farm for Education and Research Ruthe, University of Veterinary Medicine Hannover, Sarstedt, Germany; ^3^KWS LOCHOW GmbH, Bergen, Germany; ^4^Science and Innovation for Sustainable Poultry Production (WING), University of Veterinary Medicine Hannover, Vechta, Germany; ^5^Department of Nutrition and Nutritional Deficiency Diseases, Faculty of Veterinary Medicine, Mansoura University, Mansoura, Egypt

**Keywords:** turkey, rye, growth performance, litter quality, foot pad dermatitis

## Abstract

**Introduction:**

Rye is one of the most important cereal crops in Central Europe, thus attempts have been made to include it in the diets of birds to reduce production costs, since the cost of feed accounts for as much as 50 %−70 % thereof. Nevertheless, the use of rye has been limited to date, particularly in turkeys. This study aimed to test the effects of rye inclusion up to 10 % on growth, excreta, and/or litter dry matter, and foot pad health.

**Methods:**

Four trials were performed with a total of 4,322, 4,307, 4,256, and 4,280 female turkeys (BUT BIG 6, Aviagen) for trials 1, 2, 3, and 4, respectively. All birds were fed commercial starter diets for the dietary phases 1 and 2 (up to d 35 of life). Thereafter, at the start of the study, the control group received commercial supplementary feed with 5 % or 10 % wheat until the end of the fattening period. The experimental group was offered supplementary feed to which instead of wheat increasing levels of rye were added stepwise from 5 % to 10 %.

**Results:**

Using supplementary feed with rye showed no significant differences in the final body weight between the control and experimental groups (10.9 vs. 10.8 kg). The dry matter content of fresh excreta for turkeys during the experimental period did not show significant differences between both groups, except at weeks 10 and 14 of life. The feed type (either control diet or experimental diet) did not significantly affect litter dry matter content between the groups throughout the experimental period. No significant differences were noted in food pad dermatitis scoring between both groups throughout the experimental period, except at weeks 11 and 16 of life. Overall, this study showed that including proportions of rye up to 10% could replace conventional ingredients and may increase sustainability in poultry production regardless of the addition of supplementary feed.

## Introduction

The cost of feed accounts for as much as 50%−70 % of poultry production (1, 2). Due to the ongoing rise in the price of feed ingredients, producers are being forced to reconsider how best to allocate their resources for feeding efficiency (3, 4). Additionally, the International Feed Industry Federation (5) and the Food and Agriculture Organization of the United Nations (6) predict that by 2050 the production of livestock will have doubled. Therefore, it is crucial to find adequate substitutes for traditional feed sources in order to satisfy the nutritional needs of poultry. Efficient use of feedstuffs which have connections between poultry production, nutrition and a sustainable ecosystem is essential for production of sustainable human food (3, 7). One of the most relevant cereal crops in Central Europe is rye (*Secale cereale* L.). As it is affordable, simple to cultivate, and has lower soil and agro-technical needs than other cereals, rye is crucial to sustainable agriculture (7, 8). Rye has a high output yield, is resistant to fungi, tolerant to low temperatures, droughts, and unbalanced soil pH (2). In addition, rye has a small quantity of crude fiber and a lot of important proteins (9, 10). However, rye is not frequently included in poultry nutrition due to high quantities of anti-nutrients such as non-starch polysaccharides (NSP) primarily in the form of arabinoxylans, pentosans, and glucans (11). Due to the limited ability of poultry to digest these NSP, the digesta becomes more viscous, the nutrients are less digestible, and the digesta passing through the gastrointestinal tract tends to be slower (12–15). The high concentration of soluble carbohydrates in rye, which partly dissolve in the gastrointestinal tract, result in a thick, viscous solution in the digesta and cause extremely wet excreta (14, 16, 17). For environmental and economic sustainability, strategies to increase feed efficiency are especially crucial, and there are growing investments and efforts being undertaken to reduce anti-nutritional elements in wheat types (18). Due to the typically high levels of alkylresorcinols, which have been significantly reduced from over 1,000 mg kg^−1^ in the old varieties (19) to 815 mg kg^−1^ in the new ones (20), or even to 401 mg kg^−1^ in new types of hybrid rye, the amount of anti-nutritive compounds is limited and a reduction in feed palatability is no longer observed (20). This suggests that hybrid rye cultivars could be incorporated into the diet of broiler chickens, which primarily consists of cereal grains like wheat and corn (up to 65 %) in accordance with Alquaisi et al. (21). Other types of cereals, including traditional rye cultivars, are less common because of their high anti-nutrient content, which reduces nutrient conversion and digestion and, as a result, lowers performance (22). In diets supplied in mash form, there have been attempts to incorporate ground or whole rye grain in varying amounts. Recent research on the nutritional value of intact rye as a feed ingredient for broilers showed that using significant proportions of intact rye (gradual increase from 2 % at d 8 up to 20 % at d 29-d 33) could replace traditional ingredients in the chicken industry (23).

The prevalence and severity of foot pad dermatitis (FPD) are influenced by a wide range of circumstances which may affect litter quality and consequently the foot pad health. However, the most significant contributor to FPD is poor litter quality, namely moist litter (23–27). For the health and welfare of animals, it is therefore particularly important to achieve acceptable litter quality and a low prevalence of FPD. Therefore, the aim of this experiment was to determine whether rye could effectively substitute wheat in the diets of turkeys that were being raised for meat. The study also sought to determine the impact on the excreta and/or litter quality as well as foot pad health when gradually adding broken rye levels of up to 10 % to diets for growing turkeys.

## Materials and methods

The fattening turkeys in this study were raised under standardized husbandry conditions and subjected to a standard fattening procedure on the Farm for Education and Research Ruthe, University of Veterinary Medicine Hannover, Foundation, Sarstedt, Germany. Animal experiments were carried out in accordance with German regulations. Since no relevant interventions according to the Animal Protection Act (§ 7, paragraph 2, sentence 3) had been carried out on live animals, the study was not an animal experiment, and thus did not require approval from the competent authority. This has been checked by the animal welfare officer of the University of Veterinary Medicine Hannover.

### Experimental design and housing

Four trials were performed with a total of 4,322, 4,307, 4,256, and 4,280 female turkeys (B.U.T. Big 6, Aviagen Turkeys Ltd., Tattenhall, UK). These four trials (trial 1, 2, 3, and 4, respectively) together correspond to four repetitions. The birds were obtained commercially from the same hatchery in each trial (Moorgut Kartzfehn Turkey Breeder GmbH, Bösel, Germany). In all trials, the birds were divided into two groups fed either the SF with whole wheat as control diet or the SF with broken hybrid rye as experimental diet. Every group has considered as an experimental unit and the four trials were considered as the number of replications. Each group was allocated to a floor pen of 472 m^2^ in all four trials. The location of groups was swapped between the four trials to avoid any effects regarding housing conditions. Each pen was littered with wood shavings to a depth of ~4–6 cm above the concrete floor (7.60 kg/m^2^) at the start of the trials. In all groups, fresh litter was added repeatedly after the 5th week of life in identical amounts for each group (without removing the old litter). Automatic chain feeding and watering systems *via* the troughs and drinkers were used (Big Dutchman International GmbH, Vechta, Germany). The temperature (mean = 15°C during experimental period), humidity (mean = 75% during experimental period), and light in the stables were controlled automatically (ViperTouch; Big Dutchman International GmbH). The stall was heated with a gas air-heating system and controlled by an automatic control assembly for temperature and humidity.

### Diets

**Figure 1:**
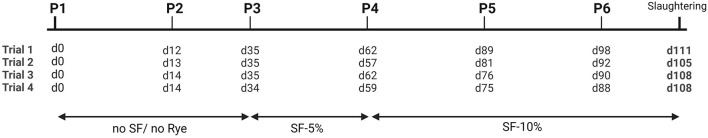
Overview of the timing of the change of feeding phases and feeding diets. P, Phase of feeding; d, day of life; SF, supplementary feed to whole wheat or broken rye; the figure was created with Biorender.com.

Feed and water were available *ad libitum* for all groups. All birds in the four trials were fed identical commercial complete diets for the first five weeks of life (P1 and P2 dietary phases). For the P3-P6 dietary phases the diets for control group and experimental group were based on the same supplementary feed (SF) for each phase. To the SF of each feeding phase whole wheat was added in the control diets and broken rye was added in the experimental diets in concentrations of 5 % (Phase 3) or 10 % (Phase 4–6) respectively (Figure 1). With the beginning of the sixth week of life (P3-P6 dietary phases), the experimental diets were fed to the experimental groups and control diets to control groups (Figure 1).

Four different types of SF were offered to the birds during the trials: SF-5 % was used from d 34 to d 58 (trial 4); d 35 to d 56 (trial 2)/d 61 (trials 1 & 3), while SF-10 % for P4 was used from d 57 (trial 2)/d 59 (trial 4)/d 62 (trials1& 3). The SF-10 % for P5 was used from d 75/ d 76/d 81/d 89 (for trials 1, 2, 3, and 4, respectively), while SF-10 % for P6 was offered from d 88/ d 90/ d 92/ d 98 (for trials 1, 2, 3, and 4, respectively) and onwards (Figure 1).

The commercial complete diets as well as pelleted SF were based on wheat grain, yellow corn, soybean meal, sunflower meal, and rapeseed meal (see Supplementary Tables 1–4 for details of feed ingredients) produced and delivered to the farm in a silo from BEST 3 Geflügelernährung GmbH, Twistringen, Germany. The broken rye (KWS Trebiano) grain was provided by KWS LOCHOW GmbH, Bergen, Germany in another silo. The SF-5 % and SF-10 % diets were formulated and 5 % or 10 % of the whole wheat (control diet) or broken rye (experimental diet) were added afterwards. For control diets the feed producer added whole wheat to the SF, while for experimental diets broken rye was mixed in the feeding lines just directly before filling the feeders. Moreover, it has to be mentioned that all diets in the four trials contained enzymes which were included in the SF (see Supplementary Tables 5–8 for details of feed analysis and composition).

### Feed analysis

The Association of German Agricultural Analytic and Research Institutes (VDLUFA) methodologies were used to determine the chemical composition of the commercial diets as well as the SF (Table 1) for the four trials (36). Weighing the samples before and after they had been dried at 103°C allowed to calculate the dry matter (DM) content. Weighing the samples prior to and following the 600°C combustion allowed the researchers to determine the crude ash content using the muffle furnace. The crude fiber content was assessed by washing the samples in diluted acids and alkalis using Foss Fibertec 2010 Hot Extractor. The crude fat content was quantified using automatic hydrolysis and extraction with Hydrotherm and Soxtherm 416 (C. Gerhardt GmbH & Co. KG, Königswinter, Germany). Additionally, the total nitrogen concentration was determined using the rapid MAX N exceed nitrogen and protein analyzer (Elementar Analysensysteme GmbH, Langenselbold, Germany). The LuffSchoorl method was employed to test the sugar levels in the samples, and atomic absorption spectrometry was utilized to analyze the minerals (Zeenit 700P, Analytik Jena GmbH, Jena, Germany). The analysis of amino acid contents was performed using ion exchange chromatography (Biochrom 30+, Biochrom Ltd., Cambridge, United Kingdom). Finally, a polarimetrical approach was used to assess the starch content of the diets (Schmidt und Haensch GmbH & Co., Berlin, Germany). All experimental diets represented typical commercial formulations, since they were designed to be isocaloric and isonitrogenous, and the essential amino acids were calculated to be almost identical between all of the diets (see Supplementary Tables 1–4 for details).

**Table 1:** Analyzed chemical composition of control diet and experimental diets for dietary phase 3–6 (mean + SD first to fourth trial).

**Item [g/kg DM]**	**P3**		**P4**		**P5**		**P6**	
	**Control (SF + 5% wheat)**	**Experimental (SF + 5% broken rye)**	**Control (SF + 10% wheat)**	**Experimental (SF + 10% broken rye)**	**Control (SF + 10% wheat)**	**Experimental (SF + 10% broken rye)**	**Control (SF + 10% wheat)**	**Experimental (SF + 10% broken rye)**
Dry matter	885 ± 7.05	883 ± 8.46	878 ± 4.03	877 ± 3.95	878 ± 3.42	877 ± 1.71	880 ± 1.00	877 ± 1.50
Crude ash	64.1 ± 1.81	64.6 ± 1.09	54.2 ± 1.18	54.4 ± 0.70	49.5 ± 3.43	52.1 ± 3.84	46.4 ± 2.78	48.4 ± 1.88
Crude fat	49.7 ± 3.36	51.5 ± 3.12	53.4 ± 5.07	54.3 ± 4.35	67.8 ± 3.77	68.5 ± 2.21	87.8 ± 5.16	87.0 ± 5.64
Crude fiber	30.7 ± 1.73	29.9 ± 2.68	31.8 ± 1.93	32.4 ± 1.99	33.5 ± 2.96	33.3 ± 2.29	32.8 ± 1.25	34.9 ± 1.87
Crude protein	263 ± 6.45	262 ± 3.32	221 ± 10.4	218 ± 9.60	196 ± 4.19	193 ± 8.34	180 ± 5.89	174 ± 4.65
Starch	426 ± 9.18	420 ± 6.00	496 ± 10.1	486 ± 4.20	507 ± 1.91	506 ± 5.83	517 ± 8.14	510 ± 11.^a^
Sugar	52.0 ± 5.94	53.8 ± 5.25	43.4 ± 3.41	48.2 ± 6.61	39.3 ± 1.38	41.5 ± 2.22	37.4 ± 2.00	39.9 ± 1.03
Calcium	11.8 ± 0.41	11.6 ± 0.39	9.80 ± 0.86	9.75 ± 0.34	8.75 ± 0.82	8.88 ± 0.96	8.30 ± 0.87	8.17 ± 0.73
Magnesium	2.23 ± 0.15	2.24 ± 0.13	1.90 ± 0.11	1.90 ± 0.11	1.80 ± 0.05	2.05 ± 0.11	1.77 ± 0.11	2.05 ± 0.10
Phosphors	7.63 ± 0.63	7.47 ± 1.11	6.56 ± 0.18	6.60 ± 0.16	6.12 ± 0.22	6.07 ± 0.31	5.65 ± 0.17	5.61 ± 0.05
Sodium	1.62 ± 0.07	1.67 ± 0.04	1.54 ± 0.20	1.64 ± 0.08	1.78 ± 0.04	1.80 ± 0.09	1.85 ± 0.10	1.85 ± 0.10
Potassium	10.1 ± 0.46	10.1 ± 0.29	7.91 ± 0.32	8.03 ± 0.26	6.66 ± 0.44	6.73 ± 0.43	6.01 ± 0.61	6.06 ± 0.55
Copper [mg/kg DM]	24.3 ± 7.00	24.6 ± 7.46	22.1 ± 7.71	24.0 ± 9.19	23.2 ± 7.43	23.2 ± 6.96	22.6 ± 2.47	23.7 ± 3.85
Zinc [mg/kg DM]	131 ± 20.9	128 ± 21.2	133 ± 14.7	99.8 ± 55.5	117 ± 12.9	118 ± 8.46	121 ± 4.57	116 ± 8.66
Iron [mg/kg DM]	293 ± 58.7	290 ± 57.5	228 ± 13.7	226 ± 13.6	215 ± 27.9	235 ± 37.3	201 ± 23.5	229 ± 18.4
Manganese [mg/kg DM]	159 ± 21.0	142 ± 21.9	145 ± 21.5	137 ± 19.5	139 ± 18.3	135 ± 16.3	125 ± 17.5	130 ± 19.2
AME_N_ [MJ/kg]	13.6 ± 0.13	13.5 ± 0.17	14.1 ± 0.45	14.0 ± 0.26	14.3 ± 0.17	14.3 ± 0.14	14.9 ± 0.15	14.8 ± 0.13
Arginine	16.9 ± 0.37	17.0 ± 0.62	13.9 ± 1.18	13.7 ± 1.04	12.1 ± 1.39	11.6 ± 1.53	10.7 ± 1.16	10.2 ± 1.28
Cysteine	4.18 ± 0.66	4.22 ± 0.59	3.73 ± 0.32	3.68 ± 0.49	3.44 ± 0.45	3.48 ± 0.50	3.37 ± 0.49	3.17 ± 0.55
Isoleucine	10.8 ± 0.45	10.8 ± 0.38	8.76 ± 0.90	8.60 ± 0.75	7.32 ± 0.30	7.06 ± 0.58	6.66 ± 0.55	6.36 ± 0.68
Leucine	18.8 ± 0.65	18.9 ± 0.26	15.6 ± 1.21	15.3 ± 1.25	13.4 ± 0.50	12.8 ± 0.80	12.4 ± 0.83	11.9 ± 1.01
Lysine	16.3 ± 0.26	16.6 ± 0.56	14.3 ± 0.90	14.2 ± 0.72	13.2 ± 0.53	12.8 ± 0.60	11.9 ± 0.28	11.5 ± 0.33
Methionine	7.06 ± 0.94	6.89 ± 0.78	6.04 ± 0.66	6.11 ± 0.53	5.71 ± 0.87	5.84 ± 0.32	5.18 ± 0.81	5.02 ± 0.68
Phenylalanine	12.5 ± 0.37	12.5 ± 0.24	10.2 ± 0.67	9.99 ± 0.62	8.57 ± 0.18	8.23 ± 0.46	7.87 ± 0.33	7.42 ± 0.41
Threonine	10.5 ± 0.80	10.6 ± 0.61	9.48 ± 0.69	9.70 ± 1.20	8.31 ± 0.81	8.01 ± 0.47	6.77 ± 0.52	7.18 ± 0.99
Valine	12.3 ± 0.50	12.3 ± 0.24	10.4 ± 0.93	10.2 ± 0.80	9.09 ± 0.40	8.83 ± 0.75	8.30 ± 0.60	8.01 ± 0.79

SF, supplementary feed for dietary phase 3–6 to whole wheat inclusion (5% or 10% respectively) for control diets or broken rye inclusion (5% or 10% respectively) for experimental diets.

P3 (d 35-61); P4 (d 62-88); P5 (d 89-97); P6 (d 98-111).

a AME_N_ (MJ/kg) = 0.01551 × g/kg crude protein+0.03431 × g/kg crude fat+0.01669 × g/kg starch+0.01301 × g/kg sugar.

### Performance parameters

Farm data, including feed and water intake, were automatically recorded in all four experiments. Additionally, the farm measured body weight (BW) every day using hanging automatic scales (Swing 70, Big Dutchman International GmbH, Vechta, Germany) that were placed in the middle of the barn (near to the ground where birds could jump over them). Weekly BW measurements were taken on digital scales (BAT 1, VEIT Electronics, Moravany, the Czech Republic) after randomly selecting 50 birds from each group and catching them. Each group's animal losses were noted. The feed conversion ratio (FCR) was calculated by dividing the total flock feed intake by the total flock weight gain during fattening. The cumulative feed intake of the dead birds was calculated to estimate the corrected FCR.

### Litter quality and foot pad dermatitis scoring

Weekly litter samples were taken from nine locations/spots in each stable for all trials to measure the DM content. Briefly, the stable was divided mathematically into nine squares then the central area of each square was chosen for collecting the samples. In order to assess the DM, nine fresh excreta samples from each group were also obtained (and pooled) on the same day and from the same areas as for litter samples. From the litter surface, only the fresh, pure excreta (free of litter debris) were collected. In accordance with Mayne et al. (24), the foot pads of the birds (50 randomly selected birds/group) were graded weekly on a scale from 0 to 7: Score 0 denoted healthy skin, whereas 7 denoted necrosis in more than 50 % of the foot pad region. For each bird, the average of the two legs' scores was calculated. In a prior study, the footpad score was demonstrated (28).

### Statistical analyses

Statistical analysis was performed using the Statistical Analysis System for Windows, the SAS^®^ Enterprise Guide^®^ version 9.3 (SAS Institute Inc., Cary, NC, USA). For all parameters, mean values as well as the standard deviation of the mean were calculated. For the individual bird's BW, data and FPD Scores the individual data of 50 random sample birds out of each stable at each time point were used. For data of water intake, feed intake, water to feed ratio, corrected feed conversion ratio (cFCR), slaughterhouse BW and mortality rate the group data of the stables were basis of the calculation. For DM content of litter and excreta, nine samples in each stable were taken according to a standardized system for statistical calculation. For all data except the FPD scores, a Shapiro-Wilk test for normal distribution was performed and normally distributed data were checked for significant differences with the Ryan-Einot-Gabriel-Welsch Test (simple ANOVA). Not normally distributed data and FPD scores were checked for significant differences with a Kruskal-Wallis test followed by a Wilcoxon two-sample test. Differences with a significance level of p ≤ 0.05 were considered significant.

## Results

The water intake did not differ significantly between the groups either during the entire fattening period (dietary phase: P1–P6) or during the experimental period (dietary phase: P3–P6) as presented in Table 2. No significant differences were noted in the feed intake between both groups in both periods (Table 2). Also, the water-to-feed intake ratio did not significantly differ between the groups either for the entire fattening period (dietary phase: P1–P6) or the experimental period (dietary phase: P3–P6).

**Table 2:** Water intake, feed intake, and water-to-feed ratio of turkeys for the four trials per bird.

**Item**	**Feed**		***P*-value**
	**Control**	**Experimental**	
Water intake [g], P1-P6	50,458 ± 2,330	51,120 ± 1,163	0.6295
Water intake [g], P3-P6	44,066 ± 2,062	44,745 ± 1,349	0.6016
Feed intake [g], P1-P6	27,235 ± 2,086	27,192 ± 1,162	0.9721
Feed intake [g], P3-P6	25,469 ± 948	25,468 ± 624	0.9987
W:F, P1-P6	1.86 ± 0.13	1.88 ± 0.08	0.7862
W:F, P3-P6	1.73 ± 0.05	1.76 ± 0.04	0.3968

P1–P6 = dietary phases; W:F = water:feed intake ratio; n = 4.

### Growth performance

Table 3 shows the average BW during the entire experimental period from the 6th week until the 17th week of life (dietary phase: P3–P6). No significant differences in BW during the experimental period were noted, except at weeks 7 and 15 of life. The average BW at the 16th week of life was 11,692 vs. 11,484 g for the control and experimental groups, respectively.

**Table 3:** Average body weight [g] of turkeys for the four trials.

**Week of life**	**Feed**		***P*-value**
	**Control**	**Experimental**	
5 (d35)	1,690 ± 192	1,718 ± 184	0.1336
6	2,497 ± 260	2,495 ± 241	0.9331
7	3,268 ± 288^b^	3,366 ± 304^a^	0.0010
8	4,321 ± 365	4,277 ± 348	0.2210
9	5,042 ± 497	5,057 ± 487	0.7560
10	6,243 ± 739	6,349 ± 777	0.1635
11	7,296 ± 769	7,290 ± 802	0.9359
12	8,350 ± 747	8,271 ± 732	0.2847
13	9,252 ± 753	9,289 ± 856	0.6425
14	10,176 ± 666	10,066 ± 747	0.1192
15	11,229 ± 725^a^	10,913 ± 804^b^	0.0004
16	11,692 ± 686	11,484 ± 823	0.1717

a, b Means in a row with different superscripts differ significantly (p < 0.05); n = 200 in week 5–14; n = 150 for week 15; n = 50 in week 16.

The performance parameters and mortalities recording by the farm as well as by the slaughterhouse for the four trials are presented in Table 4. The cFCR during the experimental period (P3–P6) did not significantly differ between the groups (2.82 vs. 2.93 for control and experimental groups, respectively). Furthermore, the final BW according to the slaughterhouse data did not differ significantly between the groups (control group=10.9 vs. 10.8 kg for the experimental group). Additionally, the mortality rate did not differ significantly between the groups either during the entire period (dietary phase: P1–P6) or during the experimental period (dietary phase: P3–P6).

**Table 4:** Corrected feed conversion ratio (cFCR), slaughterhouse BW as well as the mortality rate of turkeys for the four trials.

**Item**	**Feed**		***P*-value**
	**Control**	**Experimental**	
cFCR, P1-P6	2.515 ± 0.217	2.564 ± 0.144	0.7141
cFCR, P3-P6	2.820 ± 0.143	2.925 ± 0.088	0.2584
BW at slaughterhouse [kg]	10.930 ± 0.382	10.753 ± 0.300	0.4930
Mortality rate [%], P1-P6	1.855 ± 0.193	1.960 ± 0.693	0.7803
Mortality rate [%], P3-P6	1.118 ± 0.263	1.475 ± 0.641	0.3420

cFCR, corrected feed conversion ratio; n = 4.

### Excreta dry matter

The DM content of fresh excreta for turkeys during the experimental period did not show significant differences between the two groups, except at weeks 10 and 14 of life (Table 5).

**Table 5:** Dry matter content [%] of excreta for the four trials during the experimental period (dietary phase: P3–P6).

**Week of life**	**Feed**		***P*-value**
	**Control**	**Experimental**	
5 (d35)	21.3 ± 1.86	21.2 ± 1.86	0.7594
6	21.3 ± 1.42	21.1 ± 1.61	0.5630
7	21.1 ± 1.75	20.5 ± 1.32	0.0612
8	20.4 ± 3.26	20.6 ± 0.95	0.7921
9	21.1 ± 1.95	21.3 ± 1.30	0.5204
10	20.4 ± 1.02^b^	20.9 ± 1.06^a^	0.0318
11	21.1 ± 1.47	20.6 ± 1.52	0.2108
12	21.7 ± 1.21	21.3 ± 1.17	0.2709
13	22.3 ± 1.07	22.1 ± 1.41	0.5028
14	22.7 ± 0.94^a^	22.2 ± 1.03^b^	0.0165
15	21.9 ± 1.43	21.6 ± 1.40	0.4989
16	22.0 ± 1.21	21.7 ± 1.42	0.5628

a, b Means in a row with different superscripts differ significantly (p < 0.05); n = 36 in week 5–14; n = 27 for week 15; n = 9 in week 16.

### Litter quality

The feed type (either control diet or experimental diet) did not significantly affect the litter DM content between the groups throughout the experimental period from P3 until P6 (Table 6).

**Table 6:** Dry matter content [%] of litter for the four trials during the experimental period (dietary phase: P3–P6).

**Week of life**	**Feed**		***P*-value**
	**Control**	**Experimental**	
5 (d35)	77.6 ± 5.55	76.7 ± 5.98	0.5261
6	72.4 ± 6.47	72.5 ± 5.33	0.9117
7	69.5 ± 7.29	67.7 ± 6.45	0.6114
8	61.4 ± 10.4	61.5 ± 8.49	0.9397
9	57.4 ± 10.3	56.3 ± 9.89	0.6784
10	57.5 ± 9.14	58.4 ± 10.64	0.7134
11	54.7 ± 8.27	52.9 ± 6.29	0.3076
12	56.2 ± 10.7	55.6 ± 9.94	0.7845
13	61.6 ± 8.20	58.8 ± 8.54	0.1558
14	61.3 ± 9.55	60.2 ± 8.08	0.6003
15	57.7 ± 6.42	59.5 ± 6.70	0.3177
16	52.4 ± 5.84	53.6 ± 3.39	0.6079

n = 36 in week 5–14; n = 27 for week 15; n = 9 in week 16.

The final litter DM content or the amount of final litter in fresh/DM basis did not significantly differ between both groups (Table 7).

**Table 7:** Parameters of final litter for the four trials.

**Item**	**Feed**		***P*-value**
	**Control**	**Experimental**	**Feed**
Final litter DM [%]	58.1 ± 6.23	57.5 ± 3.50	0.8534
Amount of final litter in fresh basis [kg]	41,671 ± 2,486	43,468 ± 3,600	0.4428
Amount of final litter in DM basis [kg]	24,288 ± 3,542	24,957 ± 2,787	0.7764

The values in each item are the means of 4 analyzed samples.

### Foot pad scoring

No significant differences were noted in the FPD scoring between both groups throughout the experimental period, except at weeks 11 and 16 of life (Table 8). At week 11, the broken rye group (experimental group) showed significantly higher FPD scores compared to the control group (4.97 vs. 4.70). However, at week 16 of life, the birds in the control group showed significantly higher FPD scores compared to the broken rye group (4.96 vs. 4.65).

**Table 8:** Foot pad dermatitis scoring of turkeys for the four trials.

**Week**	**Feed**		***P*-value**
	**Control**	**Experimental**	**Feed**
5 (d35)	2.51 ± 0.93	2.38 ± 0.78	0.3989
6	3.21 ± 1.22	3.14 ± 1.10	0.3876
7	3.69 ± 1.11	3.51 ± 0.98	0.0673
8	3.74 ± 0.94	3.68 ± 0.80	0.3877
9	4.13 ± 0.84	4.25 ± 0.94	0.4230
10	4.22 ± 0.97	4.37 ± 1.12	0.2598
11	4.70 ± 0.97^b^	4.97 ± 1.25^a^	0.0254
12	5.19 ± 0.95	5.27 ± 1.01	0.3289
13	5.21 ± 0.94	5.17 ± 0.96	0.7173
14	5.36 ± 1.02	5.31 ± 0.88	0.6071
15	5.34 ± 0.93	5.49 ± 0.91	0.1170
16	4.96 ± 0.71^a^	4.65 ± 0.82^b^	0.0208

a, b Means in a row with different superscripts differ significantly (p < 0.05); n = 200 in week 5-14; n = 150 for week 15; n = 50 in week 16.

## Discussion

Maximizing the effectiveness of all feedstuffs that highlight the link between poultry production, nutrition and sustainable ecosystem services is crucial to ensure the continuity of good and sustainable contributions to a stable human food supply as well as to animal feed supply (3). Therefore, a detailed assessment of the nutritional effectiveness of new rye hybrids in poultry studies is required. Furthermore, this current study could be the first research study dealing with the addition of hybrid broken rye, particularly to the fattening turkey diet under practical field conditions (four consecutive trials).

At the 16th week of life, the average final BW (individual bird data) for the control and experimental groups was 11,692 g and 11,484 g, respectively, almost above the available B.U.T. 6 female performance objectives for turkeys of 11.29 kg (29). Moreover, in the current study, the calculated cFCR was 2.52 for the control group vs. 2.57 for the experimental group during the entire fattening period (dietary phase P1–P6). The evaluation of slaughterhouse data showed a mean daily BW gain for the rye group of 99.75 g and for the control group of 101.5 g. The monthly mean of the slaughterhouse data of comparison of all monthly slaughtered turkeys was 94 g. It seems that although the cFCR was slightly lower compared to the performance objectives for B.U.T. Big 6 female turkeys, the daily gain was higher compared to the monthly farm comparison of the slaughterhouse (29).

To the best of our knowledge, we did not find any literature regarding the effects of including broken rye in the diet of fattening turkeys on performance parameters. However, in broilers, Abd El-Wahab et al. (23) observed in two trials that the average BW (slaughterhouse data) was about 2,059 and 1,947 g for control and experimental groups, respectively at d 33 of life in the first trial, exceeding the available Ross 308 performance objectives of 1946 g (30). However, in the second trial in this previous study, this was not the case (1,883 and 1,747 g for control and experimental groups, respectively). Additionally, the corrected FCR in the first trial was identical for the two groups (1.50), whereas in the second trial, the calculated corrected FCR was 1.49 for the control group vs. 1.51 for the experimental group (23).

The results of additional studies on broilers concerning the effects of rye inclusion on broiler performance are consistent (2, 31). According to these findings, Teirlynck et al. (32) found no effect on BW of broiler diets containing 5 % rye from days 1 to 42. Similar to this, a study by Arczewska-Wlosek et al. (17) reported no detrimental effects of ground rye (20 %) on performance during the grower-finisher period in older broilers (d 22-d 42). However, according to Van Krimpen et al. (16), broiler diets containing 10 % rye from days 15 to 28 of life had a negative impact on performance when compared to diets containing 5 % rye (1,096 g for 10 % rye vs. 1,127 at day 21; 1,816 vs. 18,77 g at day 28 for 5 % and 10 %, respectively). According to Tellez et al. (332), the growth performance of broilers given a rye-based diet (58.3% rye) decreased by approximately 43 % as a result of increased intestinal viscosity. According to our results, rye should generally be introduced gradually to the diets of fattening turkeys, especially during their early fattening phase, and then gradually increased as they get older.

Feed composition as well as feed ingredients are considered to be important factors affecting excreta and litter conditions. Although the viscosity of excreta was not determined in the present study, it is well known that rye contains high levels of soluble carbohydrates, which can dissolve in the digesta, producing a thick viscous solution and consequently very wet excreta (23, 34). According to Silva and Smithard (35), birds fed rye produced a very wet excreta, this affecting the litter DM content. In the present study, the litter DM content was not affected by the inclusion of broken rye broken rye up to 10 % in the diets of fattening turkeys. Furthermore, it seems that using broken rye or its inclusion level had no impact on the excreta quality.

The mean results in terms of foot pad health (FPD scores) were better in the experimental group compared to those in the control group (4.65 vs. 4.96) at the end of the trials (16th week of life). Most of the previous studies investigated the effect of hybrid rye on foot pad health in broilers. Abd El-Wahab et al. (28) found that feeding broiler chickens either a wheat diet (control) or a SF with broken/squashed rye resulted in low FPD scores, which might be attributed to the good litter quality as well as to the comparable digesta viscosity in the wheat group.

Standing on wet litter brings the broilers' feet in constant contact with moisture and has been suggested to induce FPD (24, 27, 37). In a recent study by Abd El-Wahab et al. (23), it was observed that in the first trial, feeding SF with rye led to significantly higher FPD scores (3.80) compared to broilers fed the control diets (2.70).

However, these significant differences were not observed in the second trial, although both groups had high FPD scores (>4). Feed technology, enzyme concepts on feed ingredients, and/or stable enrichment may not have been sufficiently taken into account here (25, 38). It is still to consider that including rye in higher amounts in the diets of poultry results in an increasing intestinal viscosity (33). Consequently, when, also in the interest of environmental sustainability, higher than 10 % of rye should be included in the diets of fattening poultry, more research is needed to better understand the opportunities of new feeding technology and of enzymes as well as the type of rye (intact or broken) on excreta viscosity, litter DM content and FPD scores.

## Conclusion

The results of this study support the view that including a relative high proportion of broken rye (10 %) in diets of fattening turkeys did not affect growth performance negatively, as no relevant differences could be observed between the rye and wheat diets during the trial in the case of sample point weighing. Moreover, it seems that excreta and litter dry matter contents were not influenced by adding broken rye to diets of fattening turkeys. Consequently, the foot pad health of fattening turkeys was not affected negatively by broken rye inclusion. Therefore, the result of this study is that broken rye can be included up to 10 % in diets for turkey hens without negative effects on performance parameters and food pad health.

## Data availability statement

The original contributions presented in the study are included in the article/Supplementary material, further inquiries can be directed to the corresponding author.

## Ethics statement

Animal experiments were carried out in accordance with German regulations. Since no relevant interventions according to the Animal Protection Act (§ 7, paragraph 2, sentence 3) had been carried out on live animals, the study was not an animal experiment, and thus did not require approval from the competent authority. This has been checked by the animal welfare officer of the University of Veterinary Medicine Hannover, whose statement we sent to the editor.

## Author contributions

Conceptualization: CV, VW, and AA. Data curation, formal analysis, validation, visualization, and writing—original draft preparation: JL. Funding acquisition: CV. Investigation: JL and AA. Methodology: JL, CS, and AA. Project administration and supervision: CV and VW. Resources: RG, AF, CV, and CS. Writing—review and editing: JL, CV, RG, AF, CS, VW, and AA. All authors have read and agreed to the published version of the manuscript.
