# Supplementary material for: Effect of replacing whole wheat with broken rye as a sustainable grain in diets of fattening turkeys on growth performance, litter quality, and foot pad health
Source: Front Vet Sci. 2023 Apr 11;10:1142500. doi: 10.3389/fvets.2023.1142500 (PMC10126500; doi:10.3389/fvets.2023.1142500)
Supplement: Supplementary file 1 [file Table_1.docx]

**Supplementary Table S1.** Ingredients in control and experimental diets (Trial 1)

| Parameter | Control diets | | | | Experimental diets | | | |
| --- | --- | --- | --- | --- | --- | --- | --- | --- |
|  | P3 (SF+5% wheat) | P4  (SF+10% wheat) | P5  (SF+10% wheat) | P6  (SF+10% wheat) | P3  (SF+5% broken rye) | P4  (SF+10% broken rye) | P5  (SF+10% broken rye) | P6  (SF+10% broken rye) |
| Ingredients | | | | | | |  |  |
| Wheat | ✓ | ✓ | ✓ | ✓ | ✓ | ✓ | ✓ | ✓ |
| Soya extraction meal | ✓ | ✓ | ✓ | ✓ | ✓ | ✓ | ✓ | ✓ |
| Corn | ✓ | ✓ | ✓ | ✓ | ✓ | ✓ | ✓ | ✓ |
| Sunflower extraction meal | ✓ | ✓ | ✓ | ✓ | ✓ | ✓ | ✓ |  |
| Rapeseed extraction meal | ✓ | ✓ | ✓ | ✓ | ✓ | ✓ | ✓ | ✓ |
| Potato protein | ✓ | ✓ | ✓ | ✓ | ✓ | ✓ | ✓ | ✓ |
| Fatty acids (Soya, rapeseed, Sunflower, palm) | ✓ | ✓ | ✓ | ✓ | ✓ | ✓ | ✓ | ✓ |
| Palm fatty acids | ✓ | ✓ | - | - | ✓ | ✓ | - | - |
| Soya oil | ✓ | - | - | - | ✓ | - | - | - |
| Calcium carbonate | ✓ | ✓ | ✓ | ✓ | ✓ | ✓ | ✓ | ✓ |
| Monocalcium phosphate | ✓ | ✓ | - | ✓ | ✓ | ✓ | - | ✓ |
| Sodium bicarbonate | ✓ | ✓ | ✓ | ✓ | ✓ | ✓ | ✓ | ✓ |
| Sodium chloride | ✓ | ✓ | ✓ | ✓ | ✓ | ✓ | ✓ | ✓ |
| Nutritional-physiological additives | | | | | | |  |  |
| Vit. A, IU | 10000 | 10000 | 10000 | 10000 | 10526 | 11111 | 11111 | 11111 |
| Vit. D3, IU | 2500 | 5000 | 5000 | 5000 | 2631 | 5555 | 5555 | 5555 |
| Vit. E, mg | 200 | 160 | 60.0 | 60.0 | 210 | 177 | 66.0 | 66.0 |
| Fe, mg | 80.0 | 50.0 | 50.0 | 50.0 | 84.2 | 55.5 | 55.5 | 55.5 |
| Cu, mg | 15.0 | 15.0 | 15.0 | 15.0 | 15.7 | 16.6 | 16.6 | 16.6 |
| Zn, mg | 70.0 | 70.0 | 70.0 | 70.0 | 73.6 | 77.7 | 77.7 | 77.7 |
| Mn, mg | 100 | 100 | 100 | 100 | 105 | 111 | 111 | 111 |
| I, mg | 2.00 | 2.00 | 2.00 | 2.00 | 2.10 | 2.20 | 2.20 | 2.20 |
| Se, mg | 0.30 | 0.30 | 0.30 | 0.30 | 0.32 | 0.33 | 0.33 | 0.33 |
| L-lysin, mg | 5460 | 7410 | 9340 | 8260 | 5750 | 8230 | 10380 | 9180 |
| Zootechnical additives | | | | | | |  |  |
| Beta-Xylanase, EPU | 1500 | 2250 | 2250 | 2250 | 1578 | 2500 | 2500 | 2500 |
| Beta-Mannanase, U | 52800 | 52800 | - | - | 55578 | 58666 | - | - |
| Phytase, OTU | 500 | 500 | 500 | 500 | 526 | 555 | 555 | 555 |

SF = Supplementary feed

**Supplementary Table S2.** Ingredients in control and experimental diets (Trial 2)

| Parameter | Control diets | | | | Experimental diets | | | |
| --- | --- | --- | --- | --- | --- | --- | --- | --- |
|  | P3 (SF+5% wheat) | P4  (SF+10% wheat) | P5  (SF+10% wheat) | P6  (SF+10% wheat) | P3  (SF+5% broken rye) | P4  (SF+10% broken rye) | P5  (SF+10% broken rye) | P6  (SF+10% broken rye) |
| Ingredients | | | | | | |  |  |
| Wheat | ✓ | ✓ | ✓ | ✓ | ✓ | ✓ | ✓ | ✓ |
| Soya extraction meal | ✓ | ✓ | ✓ | ✓ | ✓ | ✓ | ✓ | ✓ |
| Corn | ✓ | ✓ | ✓ | ✓ | ✓ | ✓ | ✓ | ✓ |
| Sunflower extraction meal | ✓ | ✓ | ✓ | ✓ | ✓ | ✓ | ✓ | ✓ |
| Rapeseed extraction meal | ✓ | ✓ | ✓ | ✓ | ✓ | ✓ | ✓ | ✓ |
| Potato protein | ✓ | ✓ | ✓ | ✓ | ✓ | ✓ | ✓ | ✓ |
| Fatty acids (Soya, rapeseed, Sunflower, palm) | ✓ | ✓ | ✓ | ✓ | ✓ | ✓ | ✓ | ✓ |
| Palm fatty acids | ✓ | ✓ | - | - | ✓ | ✓ | - | - |
| Calcium carbonate | ✓ | ✓ | ✓ | ✓ | ✓ | ✓ | ✓ | ✓ |
| Monocalcium phosphate | ✓ | ✓ | ✓ | ✓ | ✓ | ✓ | ✓ | ✓ |
| Sodium bicarbonate | ✓ | ✓ | ✓ | ✓ | ✓ | ✓ | ✓ | ✓ |
| Sodium chloride | ✓ | ✓ | ✓ | ✓ | ✓ | ✓ | ✓ | ✓ |
| Nutritional-physiological additives | | | | | | |  |  |
| Vit. A, IU | 10000 | 10000 | 10000 | 10000 | 10527 | 11111 | 11111 | 11111 |
| Vit. D3, IU | 2500 | 5000 | 5000 | 5000 | 2631 | 5555 | 5555 | 5555 |
| Vit. E, mg | 200 | 160 | 60.0 | 60.0 | 210 | 177 | 66.0 | 66.0 |
| Fe, mg | 80.0 | 50.0 | 50.0 | 50.0 | 84.2 | 55.5 | 55.5 | 55.5 |
| Cu, mg | 12.0 | 12.0 | 12.0 | 12.0 | 12.6 | 13.3 | 13.3 | 13.3 |
| Zn, mg | 80.0 | 80.0 | 80.0 | 80.0 | 84.2 | 88.8 | 88.8 | 88.8 |
| Mn, mg | 120 | 100 | 100 | 100 | 126 | 111 | 111 | 111 |
| I, mg | 2.00 | 2.00 | 2.00 | 2.00 | 2.10 | 2.20 | 2.20 | 2.20 |
| Se, mg | 0.30 | 0.30 | 0.30 | 0.30 | 0.32 | 0.33 | 0.33 | 0.33 |
| L-lysin, mg | 5760 | 7910 | 9360 | 8280 | 6060 | 8800 | 10400 | 9200 |
| Zootechnical additives | | | | | | |  |  |
| Beta-Xylanase, EPU | 2250 | 2250 | 2250 | 2250 | 2368 | 2500 | 2500 | 2500 |
| Beta-Mannanase, U | 52800 | 52800 | - | - | 55584 | 58666 | - | - |
| Phytase, OTU | 500 | 500 | 500 | 500 | 526 | 555 | 555 | 555 |
| Lasalocid sodium, mg | 125 | - | - | - | 132 | - | - | - |

SF = Supplementary feed

**Supplementary Table S3.** Ingredients in control and experimental diets (Trial 3)

| Parameter | Control diets | | | | Experimental diets | | | |
| --- | --- | --- | --- | --- | --- | --- | --- | --- |
|  | P3 (SF+5% wheat) | P4  (SF+10% wheat) | P5  (SF+10% wheat) | P6  (SF+10% wheat) | P3  (SF+5% broken rye) | P4  (SF+10% broken rye) | P5  (SF+10% broken rye) | P6  (SF+10% broken rye) |
| Ingredients | | | | | | |  |  |
| Wheat | ✓ | ✓ | ✓ | ✓ | ✓ | ✓ | ✓ | ✓ |
| Soya extraction meal | ✓ | ✓ | ✓ | ✓ | ✓ | ✓ | ✓ | ✓ |
| Corn | ✓ | ✓ | ✓ | ✓ | ✓ | ✓ | ✓ | ✓ |
| Sunflower extraction meal | ✓ | ✓ | ✓ | ✓ | ✓ | ✓ | ✓ | ✓ |
| Rapeseed extraction meal | ✓ | ✓ | ✓ | ✓ | ✓ | ✓ | ✓ | ✓ |
| Potato protein | ✓ | ✓ | ✓ |  | ✓ | ✓ | ✓ |  |
| Fatty acids (Soya, rapeseed, Sunflower, palm) | ✓ | ✓ | ✓ | ✓ | ✓ | ✓ | ✓ | ✓ |
| Palm fatty acids | ✓ | - | - | - | ✓ | - | - | - |
| Soya oil | - | ✓ | - | - | - | ✓ | ✓ | - |
| Calcium carbonate | ✓ | ✓ | ✓ | ✓ | ✓ | ✓ | ✓ | ✓ |
| Monocalcium phosphate | ✓ | ✓ | ✓ | ✓ | ✓ | ✓ | ✓ | ✓ |
| Sodium bicarbonate | ✓ | ✓ | ✓ | ✓ | ✓ | ✓ | ✓ | ✓ |
| Sodium chloride | ✓ | ✓ | ✓ | ✓ | ✓ | ✓ | ✓ | ✓ |
| Nutritional-physiological additives | | | | | | |  |  |
| Vit. A, IU | 10000 | 10000 | 10000 | 10000 | 10527 | 11112 | 11112 | 11112 |
| Vit. D3, IU | 2500 | 5000 | 5000 | 5000 | 2631 | 5556 | 5556 | 5556 |
| Vit. E, mg | 200 | 190 | 90 | 90 | 210 | 211 | 100 | 100 |
| Fe, mg | 80.0 | 50.0 | 50.0 | 50.0 | 84.2 | 55.5 | 55.5 | 55.5 |
| Cu, mg | 12.0 | 12.0 | 12.0 | 12.0 | 12.6 | 13.3 | 13.3 | 13.3 |
| Zn, mg | 80.0 | 80.0 | 80.0 | 80.0 | 84.2 | 88.8 | 88.8 | 88.8 |
| Mn, mg | 120 | 100 | 100 | 100 | 126 | 111 | 111 | 111 |
| I, mg | 2.00 | 2.00 | 2.00 | 2.00 | 2.10 | 2.20 | 2.20 | 2.20 |
| Se, mg | 0.33 | 0.33 | 0.30 | 0.30 | 0.32 | 0.33 | 0.33 | 0.33 |
| L-lysin, mg | 6770 | 6670 | 7640 | 6540 | 7130 | 7420 | 8480 | 7270 |
| Zootechnical additives | | | | | | |  |  |
| Beta-Xylanase, EPU | 3000 | 3000 | 3000 | 3000 | 3158 | 3333 | 3333 | 3333 |
| Beta-Mannanase, U | 52800 | 52800 | - | - | 55584 | 58673 | - | - |
| Phytase, FYT | 1000 | 1000 | 1000 | 1000 | 1052 | 1111 | 1111 | 1111 |
| Lasalocid sodium, mg | 125 | - | - | - | 132 | - | - | - |

SF = Supplementary feed

**Supplementary Table S4.** Ingredients in control and experimental diets (Trial 4)

| Parameter | Control diets | | | | Experimental diets | | | |
| --- | --- | --- | --- | --- | --- | --- | --- | --- |
|  | P3 (SF+5% wheat) | P4  (SF+10% wheat) | P5  (SF+10% wheat) | P6  (SF+10% wheat) | P3  (SF+5% broken rye) | P4  (SF+10% broken rye) | P5  (SF+10% broken rye) | P6  (SF+10% broken rye) |
| Ingredients | | | | | | |  |  |
| Wheat | ✓ | ✓ | ✓ | ✓ | ✓ | ✓ | ✓ | ✓ |
| Soya extraction meal | ✓ | ✓ | ✓ | ✓ | ✓ | ✓ | ✓ | ✓ |
| Corn | ✓ | ✓ | ✓ | ✓ | ✓ | ✓ | ✓ | ✓ |
| Sunflower extraction meal | ✓ | ✓ | ✓ | ✓ | ✓ | ✓ | ✓ | ✓ |
| Rapeseed extraction meal | ✓ | ✓ | ✓ | ✓ | ✓ | ✓ | ✓ | ✓ |
| Potato protein | ✓ | ✓ | ✓ |  | ✓ | ✓ | ✓ |  |
| Fatty acids (Soya, rapeseed, Sunflower, palm) | ✓ | ✓ | ✓ | ✓ | ✓ | ✓ | ✓ | ✓ |
| Palm fatty acids | ✓ | - | - | - | ✓ | - | - | - |
| Soya oil |  | ✓ | - | - |  | ✓ | ✓ | - |
| Calcium carbonate | ✓ | ✓ | ✓ | ✓ | ✓ | ✓ | ✓ | ✓ |
| Monocalcium phosphate | ✓ | ✓ | ✓ | ✓ | ✓ | ✓ | ✓ | ✓ |
| Sodium bicarbonate | ✓ | ✓ | ✓ | ✓ | ✓ | ✓ | ✓ | ✓ |
| Sodium chloride | ✓ | ✓ | ✓ | ✓ | ✓ | ✓ | ✓ | ✓ |
| Nutritional-physiological additives | | | | | | |  |  |
| Vit. A, IU | 10000 | 10000 | 10000 | 10000 | 10527 | 11112 | 11112 | 11112 |
| Vit. D3, IU | 2500 | 5000 | 5000 | 5000 | 2631 | 5556 | 5556 | 5556 |
| Vit. E, mg | 200 | 190 | 90 | 90 | 210 | 211 | 100 | 100 |
| Fe, mg | 80.0 | 50.0 | 50.0 | 50.0 | 84.2 | 55.5 | 55.5 | 55.5 |
| Cu, mg | 12.0 | 12.0 | 12.0 | 12.0 | 12.6 | 13.3 | 13.3 | 13.3 |
| Zn, mg | 80.0 | 80.0 | 80.0 | 80.0 | 84.2 | 88.8 | 88.8 | 88.8 |
| Mn, mg | 120 | 100 | 100 | 100 | 126 | 111 | 111 | 111 |
| I, mg | 2.00 | 2.00 | 2.00 | 2.00 | 2.10 | 2.20 | 2.20 | 2.20 |
| Se, mg | 0.33 | 0.33 | 0.30 | 0.30 | 0.32 | 0.33 | 0.33 | 0.33 |
| L-lysin, mg | 6740 | 6740 | 7770 | 6710 | 7090 | 7490 | 8630 | 7460 |
| Zootechnical additives | | | | | | |  |  |
| Beta-Xylanase, EPU | 3000 | 3000 | 3000 | 3000 | 3158 | 3333 | 3333 | 3333 |
| Beta-Mannanase, U | 52800 | - | - | - | 55584 | - | - | - |
| Gluconase | ✓ | ✓ | ✓ | ✓ | ✓ | ✓ | ✓ | ✓ |
| Phytase, FYT | 1000 | 1000 | 1000 | 1000 | 1052 | 1111 | 1111 | 1111 |
| Lasalocid sodium, mg | 125 | - | - | - | 132 | - | - | - |

SF = Supplementary feed

**Supplementary Table S5.** Energy content, concentrations and amino acids contents of ingredients of the compound feedstuffs for fattening turkeys in the grower period in the different groups (from analyzed values, Trial 1)

| Item  [g/kg DM] | P3 (d 35-61) | | P4 (d 62-88) | | P5 (d 89-97) | | P6 (d 98-111) | |
| --- | --- | --- | --- | --- | --- | --- | --- | --- |
|  | Control (SF+5% wheat) | Experimental  (SF+5% broken rye) | Control (SF+10% wheat) | Experimental  (SF+10% broken rye) | Control (SF+10% wheat) | Experimental  (SF+10% broken rye) | Control (SF+10% wheat) | Experimental  (SF+10% broken rye) |
| Dry matter | 890 | 889 | 881 | 878 | 878 | 877 | 879 | 879 |
| Crude ash | 65.4 | 63.9 | 54.6 | 55.3 | 53.1 | 57.0 | 50.2 | 51.0 |
| Crude fat | 47.9 | 48.3 | 49.3 | 52.2 | 64.8 | 66.3 | 84.5 | 84.8 |
| Crude fiber | 29.3 | 26.6 | 29.3 | 29.7 | 29.2 | 30.0 | 31.6 | 33.2 |
| Crude protein | 269 | 264 | 220 | 215 | 197 | 196 | 174 | 170 |
| Starch | 424 | 423 | 487 | 481 | 509 | 500 | 520 | 515 |
| Sugar | 52.2 | 54.2 | 47.0 | 50.8 | 39.8 | 44.6 | 37.1 | 40.9 |
| Calcium | 12.2 | 11.6 | 9.75 | 9.83 | 9.94 | 10.1 | 9.58 | 9.20 |
| Magnesium | 2.33 | 2.28 | 1.91 | 1.90 | 1.88 | 2.20 | 1.83 | 2.11 |
| Phosphorus | 8.04 | 8.03 | 6.48 | 6.58 | 6.23 | 6.26 | 5.63 | 5.68 |
| Sodium | 1.59 | 1.62 | 1.75 | 1.75 | 1.76 | 1.80 | 1.83 | 1.83 |
| Potassium | 10.6 | 10.5 | 8.07 | 8.25 | 6.54 | 6.76 | 5.52 | 5.72 |
| Copper  [mg/kg DM] | 29.7 | 29.0 | 27.9 | 36.1 | 29.8 | 30.3 | 25.3 | 27.3 |
| Zinc [mg/kg DM] | 105 | 99.3 | 154 | 134 | 102 | 107 | 122 | 118 |
| Iron [mg/kg DM] | 379 | 366 | 215 | 206 | 255 | 288 | 236 | 256 |
| Manganese [mg/kg DM] | 139 | 120 | 176 | 129 | 153 | 147 | 144 | 153 |
| AME_N_^1^ [MJ/kg] | 13.6 | 13.5 | 13.8 | 13.8 | 14.3 | 14.2 | 14.8 | 14.7 |
| Arginine | 17.0 | 16.5 | 13.1 | 13.5 | 11.2 | 10.7 | 9.68 | 9.25 |
| Cysteine | 4.22 | 4.26 | 3.84 | 3.79 | 3.50 | 3.93 | 3.33 | 3.05 |
| Isoleucine | 11.1 | 10.7 | 8.32 | 8.78 | 7.10 | 6.79 | 6.33 | 5.94 |
| Leucine | 19.6 | 19.2 | 15.2 | 15.7 | 13.4 | 12.8 | 11.6 | 11.1 |
| Lysine | 16.1 | 15.9 | 13.7 | 14.6 | 13.9 | 13.6 | 11.9 | 11.4 |
| Methionine | 6.66 | 6.77 | 5.70 | 5.33 | 5.09 | 5.81 | 4.26 | 4.14 |
| Phenylalanine | 12.9 | 12.6 | 9.97 | 10.1 | 8.61 | 8.19 | 7.49 | 7.15 |
| Threonine | 10.4 | 9.73 | 9.80 | 8.49 | 7.52 | 7.30 | 6.32 | 6.71 |
| Valine | 12.8 | 12.5 | 9.99 | 10.4 | 8.71 | 8.41 | 7.84 | 7.59 |

SF = Supplementary feed

^1^AME_N_ (MJ/kg) = 0.1551×% crude protein+0.3431×% crude fat+0.1669×% starch+0.1301×% sugar

Enzymes in P3 and P4 phases were phytase, xylanase, and mannanase, while in P5 and P6 phases were phytase, and xylanase

**Supplementary Table S6.** Energy content, concentrations and amino acids contents of ingredients of the compound feedstuffs for fattening turkeys in the grower period in the different groups (from analyzed values, Trial 2)

| Item  [g/kg DM] | P3 (d 35-56) | | P4 (d 57-80) | | P5 (d 81-91) | | P6 (d 92-105) | |
| --- | --- | --- | --- | --- | --- | --- | --- | --- |
|  | Control (SF+5% wheat) | Experimental  (SF+5% broken rye) | Control (SF+10% wheat) | Experimental  (SF+10% broken rye) | Control (SF+10% wheat) | Experimental  (SF+10% broken rye) | Control (SF+10% wheat) | Experimental  (SF+10% broken rye) |
| Dry matter | 877 | 876 | 880 | 880 | 882 | 876 | 881 | 876 |
| Crude ash | 64.7 | 66.2 | 53.0 | 54.4 | 47.2 | 47.7 | 43.7 | 46.5 |
| Crude fat | 47.3 | 49.7 | 49.6 | 49.5 | 64.6 | 70.1 | 82.4 | 80.6 |
| Crude fiber | 32.5 | 32.1 | 34.0 | 34.5 | 34.2 | 33.8 | 31.9 | 33.7 |
| Crude protein | 267 | 264 | 215 | 212 | 190 | 185 | 178 | 171 |
| Starch | 418 | 411 | 487 | 484 | 505 | 505 | 527 | 517 |
| Sugar | 47.7 | 49.6 | 44.2 | 45.8 | 40.5 | 41.7 | 34.7 | 38.6 |
| Calcium | 11.2 | 11.4 | 9.43 | 9.54 | 8.15 | 7.77 | 7.64 | 7.53 |
| Magnesium | 2.33 | 2.34 | 2.04 | 2.04 | 1.77 | 2.06 | 1.74 | 2.14 |
| Phosphorus | 8.00 | 8.14 | 6.51 | 6.47 | 5.79 | 5.69 | 5.46 | 5.57 |
| Sodium | 1.72 | 1.71 | 1.63 | 1.66 | 1.76 | 1.68 | 1.99 | 1.98 |
| Potassium | 10.0 | 9.86 | 7.49 | 7.67 | 6.11 | 6.11 | 5.44 | 5.47 |
| Copper  [mg/kg DM] | 24.5 | 26.6 | 24.8 | 23.7 | 26.1 | 24.7 | 24.0 | 26.7 |
| Zinc [mg/kg DM] | 134 | 139 | 121 | 129 | 119 | 127 | 123 | 127 |
| Iron [mg/kg DM] | 279 | 296 | 229 | 227 | 193 | 207 | 189 | 223 |
| Manganese [mg/kg DM] | 172 | 172 | 143 | 165 | 157 | 136 | 122 | 131 |
| AME_N_^1^ [MJ/kg] | 13.4 | 13.3 | 13.7 | 13.7 | 14.1 | 14.2 | 14.8 | 14.6 |
| Arginine | 16.4 | 16.7 | 13.1 | 12.4 | 10.6 | 9.93 | 9.74 | 8.95 |
| Cysteine | 4.48 | 4.37 | 3.94 | 4.07 | 3.70 | 3.56 | 3.50 | 3.68 |
| Isoleucine | 11.0 | 11.1 | 8.41 | 7.82 | 7.03 | 6.66 | 6.17 | 5.71 |
| Leucine | 18.9 | 18.9 | 14.7 | 13.7 | 12.7 | 12.0 | 11.9 | 11.0 |
| Lysine | 16.1 | 16.4 | 13.9 | 13.3 | 13.1 | 12.4 | 11.7 | 11.1 |
| Methionine | 7.88 | 6.71 | 5.44 | 6.49 | 5.35 | 5.55 | 4.80 | 5.67 |
| Phenylalanine | 12.6 | 12.7 | 9.94 | 9.33 | 8.32 | 7.81 | 7.77 | 7.04 |
| Threonine | 11.5 | 10.5 | 8.60 | 11.2 | 7.74 | 8.22 | 6.37 | 6.02 |
| Valine | 12.4 | 12.5 | 9.92 | 9.28 | 8.82 | 8.33 | 7.84 | 7.27 |

SF = Supplementary feed

^1^AME_N_ (MJ/kg) = 0.1551×% crude protein+0.3431×% crude fat+0.1669×% starch+0.1301×% sugar

Enzymes in P3 and P4 phases were phytase, xylanase, and mannanase, while in P5 and P6 phases were phytase, and xylanase

**Supplementary Table S7.** Energy content, concentrations and amino acids contents of ingredients of the compound feedstuffs for fattening turkeys in the grower period in the different groups (from analyzed values, Trial 3)

| Item  [g/kg DM] | P3 (d 35-61) | | P4 (d 62-75) | | P5 (d 76-89) | | P6 (d 90-108) | |
| --- | --- | --- | --- | --- | --- | --- | --- | --- |
|  | Control (SF+5% wheat) | Experimental  (SF+5% broken rye) | Control (SF+10% wheat) | Experimental  (SF+10% broken rye) | Control (SF+10% wheat) | Experimental  (SF+10% broken rye) | Control (SF+10% wheat) | Experimental  (SF+10% broken rye) |
| Dry matter | 891 | 892 | 878 | 878 | 874 | 875 | 879 | 878 |
| Crude ash | 61.4 | 64.4 | 55.6 | 53.7 | 46.0 | 51.2 | 45.2 | 48.3 |
| Crude fat | 48.8 | 52.6 | 60.1 | 59.5 | 69.6 | 70.6 | 92.9 | 93.8 |
| Crude fiber | 31.8 | 32.0 | 32.1 | 32.8 | 34.9 | 34.1 | 33.5 | 37.3 |
| Crude protein | 258 | 261 | 236 | 232 | 200 | 203 | 188 | 180 |
| Starch | 439 | 423 | 507 | 486 | 507 | 505 | 508 | 493 |
| Sugar | 47.7 | 50.4 | 38.8 | 40.4 | 37.3 | 39.9 | 38.4 | 39.5 |
| Calcium | 11.8 | 12.1 | 11.0 | 10.2 | 8.31 | 8.64 | 7.90 | 8.16 |
| Magnesium | 2.26 | 2.27 | 1.87 | 1.86 | 1.77 | 1.98 | 1.87 | 2.00 |
| Phosphorus | 7.76 | 7.90 | 6.82 | 6.84 | 6.20 | 6.37 | 5.88 | 5.56 |
| Sodium | 1.57 | 1.71 | 1.30 | 1.59 | 1.77 | 1.89 | 1.77 | 1.84 |
| Potassium | 9.49 | 10.0 | 8.22 | 8.17 | 6.88 | 7.07 | 6.48 | 6.41 |
| Copper  [mg/kg DM] | 28.5 | 29.1 | 24.8 | 22.4 | 24.3 | 24.0 | 20.8 | 21.0 |
| Zinc [mg/kg DM] | 156 | 148 | 128 | 119 | 113 | 121 | 114 | 108 |
| Iron [mg/kg DM] | 259 | 265 | 247 | 237 | 199 | 211 | 190 | 216 |
| Manganese [mg/kg DM] | 144 | 137 | 131 | 121 | 123 | 111 | 102 | 106 |
| AME_N_^1^ [MJ/kg] | 13.6 | 13.6 | 14.7 | 14.3 | 14.4 | 14.5 | 15.1 | 14.8 |
| Arginine | 16.9 | 17.0 | 15.6 | 14.9 | 12.8 | 13.1 | 12.0 | 11.6 |
| Cysteine | 3.24 | 3.41 | 3.25 | 2.97 | 2.79 | 2.77 | 2.73 | 2.45 |
| Isoleucine | 10.8 | 11.1 | 10.1 | 9.56 | 7.64 | 7.92 | 7.40 | 7.23 |
| Leucine | 18.2 | 18.7 | 17.4 | 16.7 | 13.7 | 13.9 | 13.5 | 13.1 |
| Lysine | 16.5 | 17.0 | 15.6 | 14.9 | 12.7 | 13.0 | 12.3 | 11.9 |
| Methionine | 5.92 | 6.12 | 6.96 | 6.32 | 5.40 | 5.69 | 5.57 | 4.85 |
| Phenylalanine | 12.5 | 12.3 | 11.2 | 10.8 | 8.76 | 8.87 | 8.28 | 7.94 |
| Threonine | 9.57 | 10.9 | 10.2 | 10.1 | 9.19 | 8.26 | 6.96 | 7.84 |
| Valine | 12.2 | 12.3 | 11.8 | 11.2 | 9.60 | 9.93 | 9.11 | 9.08 |

SF = Supplementary feed

^1^AME_N_ (MJ/kg) = 0.1551×% crude protein+0.3431×% crude fat+0.1669×% starch+0.1301×% sugar

Enzymes in P3 and P4 phases were phytase, xylanase, and mannanase, while in P5 and P6 phases were phytase, and xylanase

**Supplementary Table S8.** Energy content, concentrations and amino acids contents of ingredients of the compound feedstuffs for fattening turkeys in the grower period in the different groups (from analyzed values, Trial 4)

| Item [g/kg DM] | P3 (d 34-58) | | P4 (d 59-74) | | P5 (d 75-87) | | P6 (d 88-108) | |
| --- | --- | --- | --- | --- | --- | --- | --- | --- |
|  | Control (SF+5% wheat) | Experimental  (SF+5% broken rye) | Control (SF+10% wheat) | Experimental  (SF+10% broken rye) | Control (SF+10% wheat) | Experimental  (SF+10% broken rye) | Control (SF+10% wheat) | Experimental  (SF+10% broken rye) |
| Dry matter | 880 | 876 | 872 | 871 | 876 | 879 | 879 | 876 |
| Crude ash | 64.8 | 63,9 | 53.4 | 54.0 | 51.7 | 52.3 | 46.6 | 47.9 |
| Crude fat | 54.6 | 55.3 | 54.4 | 55.8 | 72.3 | 66.8 | 91.5 | 88.8 |
| Crude fiber | 29.1 | 28.7 | 31.7 | 32.4 | 35.8 | 35.3 | 34.2 | 35.5 |
| Crude protein | 256 | 257 | 213 | 212 | 196 | 187 | 180 | 176 |
| Starch | 422 | 423 | 501 | 491 | 505 | 514 | 514 | 513 |
| Sugar | 60.3 | 61.1 | 43.6 | 55.8 | 39.4 | 39.9 | 39.3 | 40.5 |
| Calcium | 11.8 | 11.2 | 9.01 | 9.43 | 8.58 | 9.00 | 8.09 | 7.79 |
| Magnesium | 2.01 | 2.05 | 1.78 | 1.79 | 1.77 | 1.94 | 1.62 | 1.93 |
| Phosphorus | 6.70 | 5.81 | 6.41 | 6.52 | 6.26 | 5.97 | 5.63 | 5.62 |
| Sodium | 1.60 | 1.65 | 1.47 | 1.57 | 1.84 | 1.82 | 1.82 | 1.75 |
| Potassium | 10.1 | 9.93 | 7.87 | 8.04 | 7.11 | 6.97 | 6.59 | 6.62 |
| Copper  [mg/kg DM] | 14.3 | 13.5 | 10.7 | 13.8 | 12.6 | 13.6 | 20.2 | 19.8 |
| Zinc [mg/kg DM] | 128 | 126 | 127 | 17.0 | 133 | 116 | 124 | 110 |
| Iron [mg/kg DM] | 253 | 231 | 222 | 232 | 214 | 234 | 188 | 220 |
| Manganese [mg/kg DM] | 182 | 137 | 130 | 131 | 124 | 144 | 130 | 130 |
| AME_N_^1^ [MJ/kg] | 13.7 | 13.7 | 14.1 | 14.0 | 14.5 | 14.3 | 15.0 | 14.9 |
| Arginine | 17.3 | 17.9 | 13.8 | 14.0 | 13.6 | 12.7 | 11.3 | 10.9 |
| Cysteine | 4.77 | 4.82 | 3.89 | 3.90 | 3.78 | 3.66 | 3.90 | 3.49 |
| Isoleucine | 10.1 | 10.3 | 8.19 | 8.24 | 7.51 | 6.85 | 6.73 | 6.57 |
| Leucine | 18.3 | 18.6 | 15.2 | 15.2 | 13.8 | 12.5 | 12.4 | 12.3 |
| Lysine | 16.6 | 17.1 | 13.8 | 13.9 | 12.9 | 12.3 | 11.7 | 11.5 |
| Methionine | 7.78 | 7.97 | 6.04 | 6.31 | 7.00 | 6.29 | 6.10 | 5.40 |
| Phenylalanine | 12.0 | 12.2 | 9.73 | 9.74 | 8.59 | 8.03 | 7.92 | 7.55 |
| Threonine | 10.7 | 11.1 | 9.31 | 9.01 | 8.78 | 8.24 | 7.42 | 8.15 |
| Valine | 11.6 | 12.0 | 9.93 | 9.96 | 9.21 | 8.64 | 8.42 | 8.08 |

SF = Supplementary feed

^1^AME_N_ (MJ/kg) = 0.1551×% crude protein+0.3431×% crude fat+0.1669×% starch+0.1301×% sugar

Enzymes in P3, P4, P5 and P6 phases were phytase, xylanase, mannanase and glucanase
